# Analysis of Patient Outcomes following Curative R0 Multiorgan Resections for Locally Advanced Gastric Cancer: A Systematic Review and Meta-Analysis

**DOI:** 10.3390/jcm13103010

**Published:** 2024-05-20

**Authors:** Viorel Dejeu, Paula Dejeu, Anita Muresan, Paula Bradea, Danut Dejeu

**Affiliations:** 1 Bariatric Surgery Department, Life Memorial Hospital, Calea Grivitei 365, 010719 Bucuresti, Romania; office@doctordejeu.ro; 2 Laboratory Medicine Unit, Betania Medical Center, Menumorut 12, 410004 Oradea, Romania; 3 Surgical Oncology Department, Emergency County Hospital Oradea, Strada Gheorghe Doja 65, 410169 Oradea, Romania; amuresan@uoradea.ro (A.M.); ddejeu@uoradea.ro (D.D.); 4 Gastroenterology Unit, Betania Medical Center, Menumorut 12, 410004 Oradea, Romania; paula.bradea@betania-centrulmedical.ro; 5 Bariatric Surgery Department, Medlife Humanitas Hospital, Strada Frunzisului 75, 400664 Cluj Napoca, Romania

**Keywords:** oncology, oncologic surgery, multiorgan resection, gastric cancer, systematic review

## Abstract

**Background:** This systematic review examines the efficacy of multiorgan resection (MOR) in treating locally advanced gastric cancer (LAGC), focusing on survival outcomes, postoperative morbidity, and mortality. **Methods:** We conducted a comprehensive search of studies in PubMed, Scopus, and Embase up to November 2023, based on the PRISMA guidelines. The inclusion criteria focused on clinical trials, observational studies, case–control studies, and qualitative research, involving patients of any age and gender diagnosed with LAGC undergoing MOR aimed at R0 resection, with secondary outcomes focusing on survival rates, postoperative outcomes, and the effects of adjuvant and neoadjuvant therapies. Exclusion criteria ruled out non-human studies, research not specifically focused on LAGC patients undergoing MOR, and studies lacking clear, quantifiable outcomes. The quality assessment was performed using the Newcastle–Ottawa Scale. The final analysis included twenty studies, involving a total of 2489 patients across a time span from 2001 to 2023. Results highlighted a significant variation in median survival times ranging from 10 to 27 months and R0 resection rates from 32.1% to 94.3%. Survival rates one-year post-R0 resection varied between 46.7% and 84.8%, with an adjusted weighted mean of 66.95%. Key predictors of reduced survival included esophageal invasion and peritoneal dissemination, the presence of more than six lymph nodes, and tumor sizes over 10 cm. Nevertheless, the meta-analysis revealed a significant heterogeneity (I^2^ = 87%), indicating substantial variability across studies, that might be caused by differences in surgical techniques, patient demographics, and treatment settings which influence survival outcomes. **Results**: The review underlines the important role of achieving R0 resection status in improving survival outcomes, despite the high risks associated with MOR. Variability across studies suggests that local practice patterns and patient demographics significantly influence results. **Conclusions:** The findings emphasize the need for aggressive surgical strategies to improve survival in LAGC treatment, highlighting the importance of achieving curative resection despite inherent challenges.

## 1. Introduction

The prognosis of locally advanced gastric cancer (LAGC) remains a formidable challenge in the field of oncological surgery [1,2]. Despite advancements in preoperative chemotherapy and radiation therapy, the survival rates post-surgery have shown only modest improvements over the past decades, with approximately 40% overall survival in all gastric cancers, and from 0% to 15% in disseminated and LAGC [3,4,5]. A critical determinant of long-term survival in patients with LAGC is the achievement of R0 resection, defined as the complete removal of the tumor with no microscopic residual disease [6]. Moreover, R0 curative resections often necessitate multivisceral resection (MVR) for LAGC, commonly associated with bloc removal of the stomach along with adjacent involved organs to achieve an R0 status [7,8,9].

Gastric cancer, as a global health issue, ranks as the fifth most common malignancy and the third leading cause of cancer-related deaths worldwide [10,11]. According to the World Health Organization, over one million new cases of gastric cancer were diagnosed in 2020, with an estimated 769,000 deaths [12,13]. The high mortality rate associated with gastric cancer is largely attributed to the late presentation of the disease, where a significant proportion of patients are diagnosed at an advanced stage. In such cases, MVR offers a potentially curative approach, albeit with increased surgical risks and the need for comprehensive postoperative care [14].

The rationale behind multivisceral resection for LAGC stems from the aggressive nature of gastric cancer, which often invades adjacent structures such as the spleen, pancreas, colon, and liver [15,16]. Traditional surgical approaches that aim for tumor removal without addressing the potential spread to adjacent organs may result in suboptimal outcomes. MVR, on the other hand, aims to improve the odds of achieving an R0 resection by extending the surgical margins beyond the stomach to include any organs that might be involved [17]. This aggressive surgical strategy has been the subject of much debate, with concerns about increased morbidity and mortality rates juxtaposed against the potential for improved survival outcomes [18].

This systematic review seeks to elucidate the survival outcomes of patients undergoing MVR for LAGC with the intent of achieving R0 resection. By compiling and analyzing data from various studies, this review aims to provide a comprehensive overview of the current evidence on the efficacy of this surgical approach. The review encompasses studies that report on overall survival (OS), disease-free survival (DFS), postoperative morbidity, and mortality rates, offering insights into the benefits and risks associated with MVR in the treatment of LAGC.

## 2. Materials and Methods

### 2.1. Eligibility Criteria 

The review considered studies for the final analysis based on the following inclusion criteria: (1) Study population: Studies must involve patients diagnosed with locally advanced gastric cancer undergoing multiorgan (or multivisceral) resection aimed at achieving R0 (curative) resection status. This includes patients across all age groups and both genders; (2) Focus on surgical outcomes and survival rates: Research must explicitly examine the survival outcomes following multiorgan resection for gastric cancer, with particular emphasis on R0 resection status. This encompasses studies assessing immediate and long-term survival rates, postoperative morbidity and mortality, associated or not with adjuvant and neoadjuvant therapy outcomes; (3) Types of studies: The review will include a broad array of study designs, such as randomized controlled trials, observational studies, clinical trials, cohort studies, case–control studies, and cross-sectional studies. Qualitative studies providing in-depth insights into patient experiences and outcomes post-resection will also be considered; (4) Outcome measures: Studies that utilize validated instruments or clearly defined parameters to assess survival rates, and postoperative complications; (5) Language: Only peer-reviewed articles published in English will be included to ensure the feasibility of thorough review and analysis.

The exclusion criteria comprised: (1) Non-human studies: Research not involving human participants, such as in vitro or animal model studies on gastric cancer, will be excluded to focus solely on human patient experiences and outcomes; (2) Broad cancer focus: Studies not specifically examining patients with LAGC undergoing multiorgan resection, or those that do not differentiate the impact of this specific surgical intervention on survival rates will be excluded; (3) Lack of specific outcomes: Studies that do not provide clear, quantifiable outcomes related to survival rates, and postoperative complications, or lack sufficient detail for a comprehensive analysis, will be excluded; (4) Grey literature: To maintain the credibility and reliability of the data included in the review, grey literature, including non-peer-reviewed articles, preprints, conference proceedings, general reviews, commentaries, and editorials, will be excluded.

### 2.2. Information Sources

To conduct a thorough and exhaustive review of the literature on survival rates following R0 curative resections after multiorgan (or multivisceral) resection for locally advanced gastric cancer, this study adopts an extensive search strategy across key electronic databases, including PubMed, Scopus, and Embase. The literature search was targeted to include publications up to 23 November 2023, capturing the most recent and relevant studies on the topic. The primary objective of the search strategy was to collect studies that evaluate survival outcomes, surgical techniques, patient demographics, and postoperative care associated with multiorgan resection in treating LAGC.

### 2.3. Search Strategy

The search strategy utilizes an expansive array of keywords and phrases pertinent to the study’s objectives, focusing on the nuances of surgical management and survival outcomes in gastric cancer. Key search terms include: “locally advanced gastric cancer”, “gastric neoplasms”, “multiorgan resection”, “extended resection”, “multivisceral resection”, “R0 resection”, “curative resection”, “palliative resection”, “survival rate”, “surgical outcomes”, “oncological outcomes”, “patient survival”, “gastric cancer surgery”, “postoperative complications”, “adjuvant therapy”, “neoadjuvant therapy”, “chemotherapy”, “radiotherapy”, “surgical morbidity”, “long-term survival”, “cancer mortality”, “disease free survival”, “prognostic factors”, “T4 cancer”, “aggressive surgery”, and “gastric carcinoma.”

To ensure a comprehensive and efficient literature retrieval, Boolean operators (AND, OR, NOT) were employed to effectively combine and refine MeSH terms. The search string included the following: ((“locally advanced gastric cancer” OR “gastric neoplasms”) AND (“multiorgan resection” OR “multivisceral resection” OR “extended resection”) AND (“R0 resection” OR “curative resection” OR “palliative resection”) AND (“survival rates” OR “surgical outcomes” OR “patient survival” OR “mortality”) AND (“postoperative complications” OR “morbidity”) AND (“chemotherapy” OR “radiotherapy”) AND (“disease-free survival” OR “long-term survival” OR “prognostic factors” OR “risk factors”)).

### 2.4. Selection Process

In accordance with the Preferred Reporting Items for Systematic Reviews and Meta-Analyses (PRISMA) guidelines [19], our selection process involved a structured and transparent method to ensure the reproducibility of our research. Initially, all retrieved records were independently screened by two reviewers to determine their eligibility based on the predefined inclusion and exclusion criteria. Discrepancies between reviewers were resolved through discussion or, if necessary, consultation with a third reviewer. We utilized automation tools to manage and track the screening process, enhancing efficiency and reducing manual errors. The review protocol and its detailed selection methodology have been registered and are openly accessible on the Open Science Framework (OSF) with the registration code osf.io/p4ebv, ensuring transparency of our research process and findings.

### 2.5. Data Collection Process

The data collection process for this systematic review commenced with the removal of duplicate entries, followed by a rigorous screening of abstracts by two independent reviewers to assess each study’s relevance based on predefined inclusion and exclusion criteria. Discrepancies between reviewers were resolved through discussion or, if necessary, consultation with a third reviewer to achieve consensus. The initial database search yielded the total number of articles that were evaluated and identified for inclusion in the final study.

### 2.6. Data Items

In our systematic review, we sought data on several outcomes related to multiorgan resection for locally advanced gastric cancer, focusing specifically on R0 resection status. The outcomes included survival rates at various time points (e.g., 1 year, 3 years, 5 years), postoperative morbidity, and mortality rates. We also analyzed the variability in these outcomes across different studies, ensuring that all results compatible with each outcome domain were captured. To decide which results to collect, we prioritized validated outcome measures and clearly defined parameters assessing survival and postoperative complications. 

Additionally, we collected data on a variety of other variables to provide a comprehensive analysis of the intervention characteristics and context. These included study characteristics (country, study year, design, and quality), patient demographics (age, gender distribution), and details of the surgical intervention. We also noted the presence of adjuvant and neoadjuvant therapies as well as the extent of lymph node involvement and tumor size, which were critical in understanding the scope and efficacy of the surgical strategies employed. Assumptions were made about missing or unclear information based on the context provided by studies with similar settings or methodologies, ensuring consistency in data interpretation across the review.

In this systematic review, LAGC was defined as gastric cancer that has invaded beyond the muscularis propria (T3) or has penetrated the visceral peritoneum (T4a) or adjacent structures (T4b), as per the TNM classification system by the American Joint Committee on Cancer (AJCC). Cases of distant metastases were excluded, with the exception of tumoral expansion with local invasion of adjacent organs. This definition identifies patients who, despite the advanced nature of their disease, may still be candidates for potentially curative surgical interventions.

Multiorgan resection or multivisceral resection is defined within the context of this review as the surgical removal of the primary gastric tumor along with one or more adjacent organs or structures to achieve a margin-negative (R0) resection. This surgical approach is indicated for locally advanced gastric cancer where the tumor’s invasion into adjacent organs necessitates their removal to ensure the complete excision of cancerous tissue. MOR aims to achieve an R0 resection status, characterized by the absence of microscopic residual tumor at the resection margins. The significance of achieving R0 resection lies in its association with improved survival outcomes, making it a crucial objective of MOR in the management of locally advanced gastric cancer.

### 2.7. Risk of Bias and Quality Assessment

For the systematic assessment of study quality and determination of risk of bias within the included studies, our review employed a dual approach, integrating both qualitative and quantitative evaluation methods. Initially, the quality of observational studies was evaluated using the Newcastle–Ottawa Scale [20], a widely recognized tool that assesses three critical dimensions: the selection of study groups, the comparability of these groups, and the ascertainment of either the exposure or outcome of interest for case–control or cohort studies, respectively. Each study is awarded stars in these categories, cumulating in a score that classifies the study quality as either low, medium, or high. To ensure the objectivity and reproducibility of our quality assessment process, each study was independently evaluated by two researchers. Discrepancies in quality assessment scores were resolved through discussion, or if necessary, consultation with a third researcher.

### 2.8. Synthesis Methods

In this systematic review, we integrated findings from selected studies through a qualitative synthesis, given the variability in study designs and outcome measures reported. The selection of studies for synthesis was based on their alignment with predefined inclusion criteria, focusing on R0 resection status and its impact on survival rates, postoperative morbidity, and mortality. To prepare data for synthesis, we performed a tabulation of survival outcomes, surgical success rates, and complication rates, while handling missing data by noting their absence and acknowledging potential impacts on our findings. Results from individual studies were summarized and presented descriptively, comparing survival outcomes and surgical effectiveness across diverse geographic and clinical settings. This approach allowed for a comprehensive conclusion about the effectiveness and risks of multiorgan resection for locally advanced gastric cancer without employing statistical meta-analysis.

A meta-analysis was conducted to evaluate the one-year survival rates of patients undergoing R0 resections for locally advanced gastric cancer. We utilized a random effects model to account for the expected variability across different studies. Survival rates were treated as proportions, and inverse variance weights were calculated for each study to determine a weighted mean survival rate. The between-study variance (T^2^) was estimated using the DerSimonian method, which adjusts the weights of individual studies to incorporate both within-study and between-study variance. Heterogeneity among study results was quantified using the I^2^ statistic, which describes the percentage of total variation across studies that is due to heterogeneity rather than chance. A high I^2^ value indicates substantial variability among the studies. All analyses were performed using standard statistical software, ensuring that all estimates were accompanied by 95% confidence intervals to assess the precision of the findings.

## 3. Results

### 3.1. Study Selection and Study Characteristics

**Figure 1:**
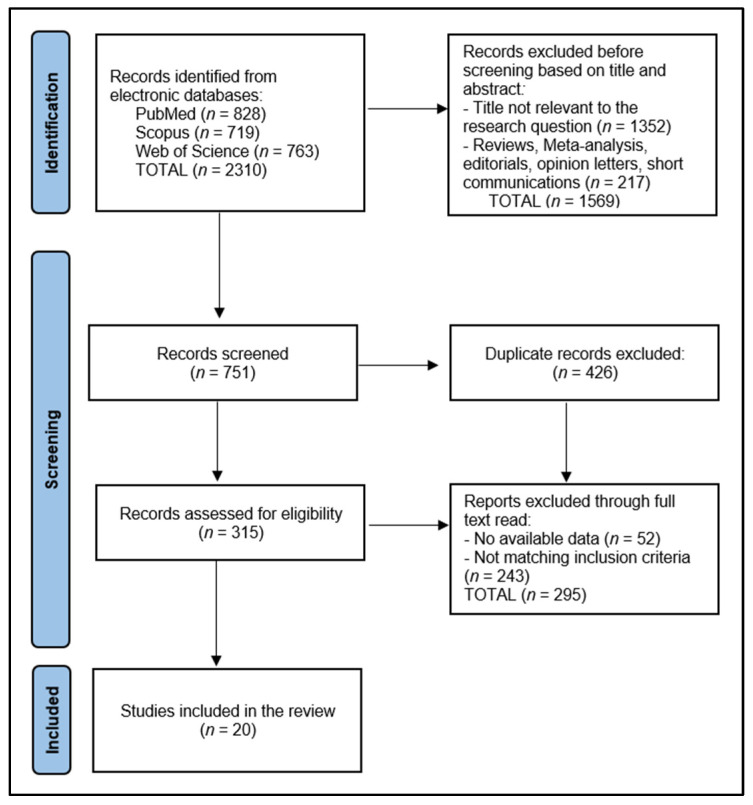
PRISMA flow diagram.

A total of 2310 articles were identified according to the initial search, of which 426 duplicate entries were eliminated, 1569 records were excluded before screening based on title and abstract, and 295 articles were excluded after full read for not matching the inclusion criteria or having no available data. The systematic review included a total of 20 studies in the final analysis [21,22,23,24,25,26,27,28,29,30,31,32,33,34,35,36,37,38,39,40], delineated in Figure 1, spanning a period from 2001 to 2023, with a geographical distribution across Asia (Japan, South Korea, China, Taiwan), Europe (Italy, Poland, Bulgaria), and South America (Brazil). The studies predominantly employed retrospective cohort designs, with seven studies (Carboni et al. [24], Jeong et al. [27], Cheng et al. [28], Li et al. [32], Mita et al. [33], Aversa et al. [37], and Zhang et al. [38]) utilizing prospective cohort approaches, indicating a varied methodological approach to investigating survival rates in R0 curative resections following multiorgan resection for locally advanced gastric cancer.

The quality of the studies varied, with a notable distinction between retrospective and prospective designs. Prospective cohorts from Italy (Carboni et al. [24], Pacelli et al. [30], Aversa et al. [37]), Taiwan (Cheng et al. [28]), South Korea (Jeong et al. [27]), and China (Li et al. [32], Zhang et al. [38]) were generally rated high in quality, reflecting the rigorous data collection and follow-up processes inherent in prospective research. Conversely, retrospective cohorts predominantly received medium quality ratings, except for a few from South Korea (Kim et al. [25]) and China (Wang et al. [26], Xiao et al. [31]), which were rated low. This variability in study quality underscores the challenges in ensuring consistency and reliability in retrospective analyses, often due to the retrospective nature of data collection and potential biases.

Japan emerged as a significant contributor to the literature on this topic, with multiple studies spanning over a decade (Dhar et al. [21], Kobayashi et al. [22], Kunisaki et al. [23], Mita et al. [29], Mita et al. [33]), reflecting a sustained interest and expertise in the surgical management of locally advanced gastric cancer within the country. These studies, alongside those from China (Wang et al. [26], Xiao et al. [31], Xiao et al. [34], Yang et al. [35], Zhang et al. [38]), South Korea (Kim et al. [25], Jeong et al. [27]), and Italy (Carboni et al. [24], Pacelli et al. [30], Aversa et al. [37]), provide a comprehensive insight into the practice patterns, surgical outcomes, and evolving trends in the management of this disease across different healthcare settings and populations.

The use of retrospective cohort designs in the majority of the studies (Dhar et al. [21], Kobayashi et al. [22], Kunisaki et al. [23], Kim et al. [25], Wang et al. [26], Mita et al. [29], Xiao et al. [31], Xiao et al. [34], Yang et al. [35], Dias et al. [36], Bobrzyński et al. [39], Vladov et al. [40]) suggests a reliance on existing medical records and databases for data collection, which, while practical for large sample sizes and long-term outcomes, may introduce recall or selection biases. The prospective cohorts (Carboni et al. [24], Jeong et al. [27], Cheng et al. [28], Li et al. [32], Mita et al. [33], Aversa et al. [37], Zhang et al. [38]), however, provide a more controlled observation of patient outcomes over time, contributing valuable longitudinal data on survival rates post-multiorgan resection, as presented in Table 1.

### 3.2. Results of Individual Studies

The analysis of patient characteristics from Table 2, involving 20 studies on locally advanced gastric cancer treated with multiorgan resection aiming for R0 curative outcomes, encompasses a total of 2489 patients. These studies spanned from 2001 to 2023, reflecting a broad international effort, with research conducted in countries including Japan, Italy, South Korea, China, Taiwan, Brazil, Poland, and Bulgaria.

For instance, Dhar et al. [21] reported a mean age of 62.5 years, while the oldest average age was noted in the study by Mita et al. [33] at 69.7 years, showcasing the variability in patient age groups across different regions and time periods. Gender distribution across the studies showed a consistent male predominance, reflective of the global epidemiology of gastric cancer. Notably, Zhang et al. [38] reported the highest percentage of R0 resection (94.3%), highlighting the potential for significant variability in surgical success rates across different healthcare settings and surgical teams.

The follow-up times and mean survival rates provided insights into the postoperative outcomes, with follow-up periods ranging from 13 months in Carboni et al. [24] to 5 years in Li et al. [32], and up to 101 months in the study by Zhang et al. [38]. These follow-up durations are crucial for understanding the long-term survival and efficacy of R0 resections in the context of multiorgan resection for locally advanced gastric cancer.

Gender distribution varied, with studies like Kobayashi et al. [22] showing a higher proportion of men (70.7%) compared to women (29.3%), a trend that was consistent across most studies, suggesting a gender disparity in the incidence of the disease requiring such extensive surgical intervention. The percentage of R0 resections achieved in these studies ranged widely, from 32.1% in Wang et al. [26] to the remarkable 94.3% in Zhang et al. [38], reflecting differences in surgical expertise, patient selection, and possibly tumor characteristics across different regions and institutions.

The staging and histology of the disease uniformly emphasized the advanced nature of gastric cancer among the patients, with a significant majority presenting with T4 gastric carcinoma. For instance, Dhar et al. [21] and Kunisaki et al. [23] reported 100% of their patient cohorts having T4 gastric carcinoma, illustrating the aggressive and advanced stage of the disease in these populations. The varied mean tumor sizes, as reported by Kobayashi et al. [22] with sizes of 9.0 cm and 10.8 cm for T3 and T4 stages, respectively, and by Jeong et al. [27] with a mean size of 7.9 cm for T2–3 and T4 stages, further highlight the significant tumor burden these patients bear.

Surgical interventions extended beyond gastrectomy to include pancreatectomy, splenectomy, liver resection, and even adrenalectomy in some cases, indicating the necessity for extensive surgical approaches to achieve R0 resection. Kobayashi et al. [22], for instance, detailed a comprehensive range of additional surgeries, including pancreatectomy and splenectomy in 43.9% of cases, showcasing the complexity of surgical management in these patients.

Complication rates varied widely across the studies, with post-operative complications ranging from as low as 9.6% in Xiao et al. [34] to as high as 75% in Bobrzyński et al. [39], indicating the high-risk nature of these extensive surgical procedures. Post-operative death rates also varied, underscoring the serious risk associated with such aggressive treatment approaches. Adjuvant treatment was a common follow-up to surgery, with many studies reporting high rates of adjuvant therapy use. Dhar et al. [21] reported adjuvant therapy in 92.7% of cases, while Xiao et al. [34] noted a lower rate of 69.3% for adjuvant therapy, as described in Table 2.

### 3.3. Results of Synthesis

**Figure 2:**
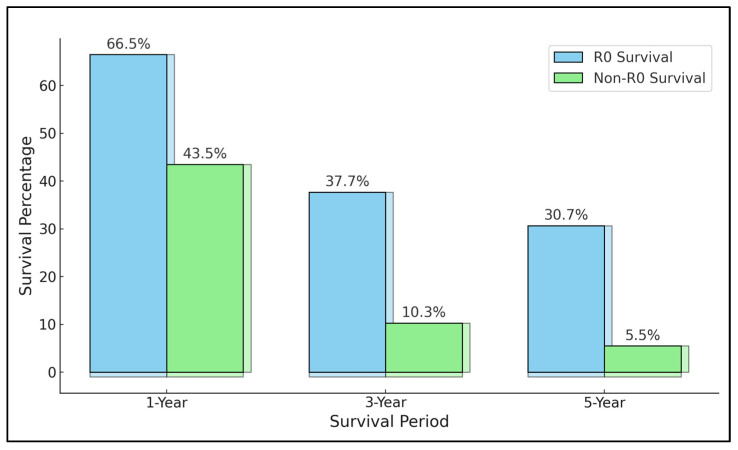
Aggregate survival rates by oncological margin.

A key finding across the studies was the variance in survival rates post R0 resection, highlighting the complex nature of treating locally advanced gastric cancer. For instance, Dhar et al. [21] reported survival rates of 46.7% at one year, 25.1% at three years, and 16.8% at five years, illustrating the challenging prognosis for patients even after achieving R0 resection. Similarly, Kobayashi et al. [22] and Kunisaki et al. [23] presented five-year survival rates of 36.9% and 32.2%, respectively, underscoring the long-term survival challenges. The aggregate survival rate was 30.7% in R0 patients compared with 5.5% in R1/R2 cases (Figure 2). These survival rates reflect the aggressive nature of the disease and the critical role of comprehensive surgical and adjuvant treatment strategies.

The contrast between R0 survival and non-curative survival rates further emphasized the importance of achieving R0 resection. For example, Cheng et al. [28] reported a significant drop in survival for patients with non-curative resections, with one-year survival at 60.0% for R0 resections compared to 28.0% for non-curative, demonstrating the stark difference in outcomes based on resection status.

The analysis also highlighted several significant risk factors for mortality, providing valuable insights into patient management and prognosis. Notably, the presence of esophageal invasion, as reported by Dhar et al. [21], and peritoneal dissemination, as noted by Kobayashi et al. [22], were associated with poorer survival rates, indicating the importance of these factors in patient selection and treatment planning. Similarly, Li et al. [12] and Mita et al. [13] identified N3 lymph node involvement as a critical risk factor, emphasizing the role of comprehensive nodal dissection and accurate staging in improving patient outcomes.

**Figure 3:**
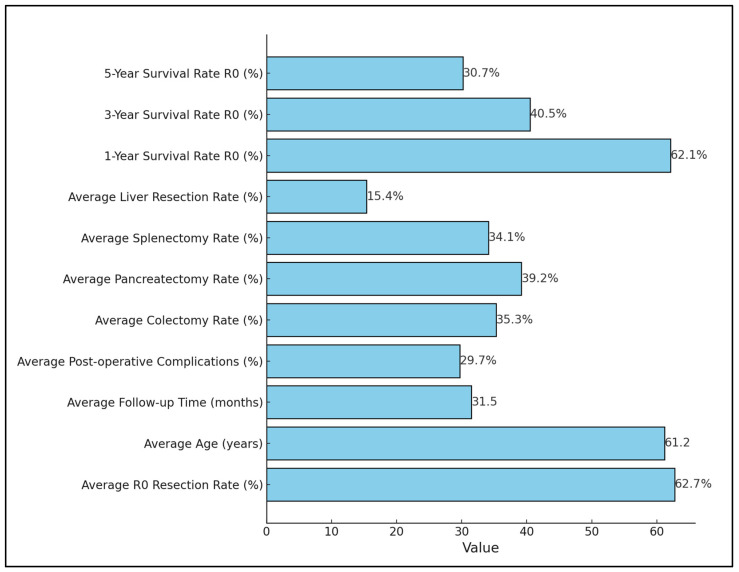
Aggregate mean values recorded through the analyzed studies.

The studies collectively concluded that aggressive surgery with curative intent, including multiorgan resection, significantly improves survival rates for patients with locally advanced gastric cancer. For instance, Jeong et al. [27] and Xiao et al. [31] highlighted the survival benefits of R0 resection, with adjuvant therapy playing a pivotal role in enhancing outcomes, as presented in Table 3 and Figure 3. Moreover, the need for a meticulous surgical approach to manage postoperative complications and ensure the best possible long-term outcomes was evident, as indicated by the high complication rates and the nuanced survival benefits observed across the studies.

Our meta-analysis of one-year survival rates for locally advanced gastric cancer patients undergoing R0 resections incorporated data from multiple studies and used a random effects model due to anticipated heterogeneity. The adjusted weighted mean survival rate was calculated at approximately 66.92%. The analysis revealed significant heterogeneity (I^2^ = 87%), indicating substantial variability in outcomes across studies. This high heterogeneity suggests that factors such as differences in surgical techniques, patient demographics, and treatment settings might influence survival outcomes.

## 4. Discussion

### 4.1. Summary of Evidence

The systematic review underscores critical insights regarding R0 curative resections following multiorgan resection for LAGC. The data reveal a demographic skew towards older males, with the majority of patients in their sixth decade. This demographic detail suggests a targeted subgroup potentially benefiting most from aggressive surgical interventions. The wide range in R0 resection rates (32.1% [26] to 94.3% [38]) across studies highlights the variability in achieving curative outcomes, possibly reflecting differences in surgical expertise, tumor characteristics, and patient selection criteria, that finally influence oncological success [41].

The advanced stage of disease in patients, primarily those with T4 gastric carcinoma, necessitates multiorgan resections, including pancreatectomy, splenectomy, and liver resection, to attempt R0 status. The notable incidence of post-operative complications, reported as high as 75% in some studies [39], underscores the significant morbidity risk these extensive procedures carry. However, it depends on how the complications are assessed and measured, as the included studies date back to as far as 2001 [21], and standardized classifications such as the Clavien–Dindo scale were not yet developed or largely accepted [42]. Concurrently, the application of adjuvant treatments across several studies suggests a shift towards a more holistic disease management approach, integrating surgical and adjuvant therapies.

The analysis distinctly illustrates the survival advantage conferred by R0 resections. Consistent improvements in survival rates post-surgery for patients achieving R0 status highlight its critical role in managing LAGC. Moreover, specific factors such as lymph node involvement, tumor size, and peritoneal or nerve invasion emerge as influential in survival outcomes, underscoring the importance of comprehensive pre-surgical planning and the potential impact of achieving an R0 resection. It was observed among studies that even though R0 status was achieved, lymph node involvement was a determining factor for survival. As such, N3 lymph node involvement and as low as 6–7 positive nodes were considered significant worsening factors for survival in many of the analyzed studies [23,25,27,28,30,32,33,35,36,40].

Moreover, these findings reveal a nuanced perspective on MOR’s role. While associated with significant survival benefits when resulting in R0 resection, the approach is also linked to high complication rates and morbidity, such as in the study by Vladov et al. [40]. This complexity necessitates a judicious, individualized approach to surgical decision-making, balancing the potential for curative resection against the inherent risks and the patient’s health status.

One of the least analyzed types of gastric cancer among the analyzed studies was the LAGC involving the gastroesophageal junction. The study by Narayan et al. [43] underscores the shift towards perioperative combination chemotherapy or neoadjuvant chemoradiation for localized disease management, the pivotal role of staging laparoscopy in enhancing staging accuracy, and the consensus favoring D2 lymph node dissection as evidenced by the 15-year follow-up results of the Dutch randomized trial [44]. It was observed that many surgical approaches emerged, such as pylorus-preserving distal gastrectomy for select early-stage gastric cancers and the selective use of multi-visceral resections and cytoreductive surgery with hyperthermic intraperitoneal chemotherapy for locally advanced tumors, marking significant strides in the field’s ongoing development. Other studies have also focused their attention on D2 dissections, such as the one by Lorenzon et al. [45], where researchers attempted a significantly greater lymph node harvest compared to D1-plus, with a mean of 31.2 versus 27.2 nodes (*p* = 0.04). Despite similar distribution between the groups, D2 dissection was found to independently correlate with improved disease-free survival, as indicated by hazard ratios highlighting the impact of D1-plus dissections on survival outcomes (HR 2.1; 95% CI 1.26–3.50). Another study by Pareekutty et al. [46] reports that D2 lymphadenectomy, when combined with R0 resection, offers acceptable morbidity and comparable survival rates to Western data, with 5-year overall survival and disease-free survival rates at 34.9% and 37.6%, respectively. Both studies underline that D2 lymphadenectomy, despite its complexity, can significantly influence long-term outcomes in patients, underscoring its potential therapeutic benefit in the comprehensive management of gastric cancer.

One of the regions that were not covered in this review is India. One proceeding publication by Deshpande et al. [47] presents a nuanced exploration of the outcomes associated with extended MOR for LAGC, a subject mired in controversy regarding its prognostic and survival benefits. The study reports a perioperative morbidity rate of 25% and a mortality rate of 5.5%, alongside a median survival time of 28 months. Notably, the survival benefit, influenced by achieving R0 or curative resection, did not reach statistical significance contrary to the majority of the studies included in this review. Furthermore, the study highlights the issue of overstaging, observed in 50% of the patients, suggesting that when the extent of organ invasion is uncertain, surgeons might still opt for en bloc resection, emphasizing the feasibility of extended multiorgan resection provided that an R0 resection is attainable. 

Another concluding thought is that men were more commonly affected by LAGC necessitating MOR. This might be attributed to genetic, environmental, and habitual factors, but other forms of locally advanced cancers, such as colon cancer were observed in a higher prevalence in women. The study conducted by Gezen et al. [48] evaluated the outcomes of MOR for locally advanced colorectal cancers, particularly focusing on clinical and pathological T4 tumors. The research found that out of 354 patients, 25.4% underwent MOR with an en bloc R0 resection achieved in 91.1% of cases. Interestingly, only a third of these clinical T4 tumors had actual adjacent organ invasions, indicating a potential discrepancy between clinical and pathological staging. The study also notes that clinical T4 tumors were more prevalent in women and were more likely to actually invade adjacent organs when located in the colon. Despite the increased operation time, bleeding, and transfusion requirements associated with multivisceral resections, the study reports no significant difference in hospital stay, complications, 30-day mortality rates, or 5-year survival rates compared to single organ resections. 

While two other systematic reviews [7,49] had a central focus on MVR in the context of gastric cancer, previous conclusions that were published more than fifteen years ago might be outdated in the context of advancements in oncology, such as the study by Brar et al. [7]. Another important study by Schizas et al. [49] had a main focus on the overall survival and complications after LAGC, identifying that the spleen, colon, and pancreas were most often removed. The leading postoperative issues included pancreatic fistulae (10.08%), intraabdominal abscesses (9.92%), and anastomotic leaks (8.09%). Survival rates were estimated at 62.2% after one year, 33.05% after three years, and 30.21% after five years for the studied group. Therefore, the current systematic review brings an exceptionally important novelty by specifically focusing on the multivisceral R0 resections in comparison to the non-curative resections in terms of 5-year long-term survival and surgical outcomes. 

Nevertheless, the current guidelines [50] recommend that all patients with LAGC receive neoadjuvant treatment before surgery to improve surgical outcomes and overall survival. However, not all patients included in our review received such treatment, which may have introduced variability in the survival outcomes and postoperative morbidity reported across the studies. This inconsistency in preoperative treatment aligns with clinical practices in different regions and underlines a critical gap in adhering to standard treatment protocols. The inclusion of patients who did not receive neoadjuvant therapy in some studies could potentially skew the results towards poorer outcomes, reflecting the natural progression of more advanced disease at the time of surgery. Future research should aim to stratify results based on the receipt of neoadjuvant treatment to provide clearer insights into its impact on the efficacy of multiorgan resection for LAGC. 

### 4.2. Limitations

The systematic review’s comprehensive analysis of survival outcomes post-multiorgan resection for locally advanced gastric cancer, while insightful, encounters several limitations that merit consideration. Firstly, the study’s dependency on retrospective cohorts introduces inherent biases, potentially affecting the reliability and generalizability of findings. The variability in patient demographics, such as age and gender distribution across studies, and the wide range of R0 resection rates reported, from 32.1% in Wang et al. [26] to 94.3% in Zhang et al. [38], underscores the heterogeneity in surgical success and patient selection criteria among different healthcare settings. Moreover, the reported survival rates and significant risk factors for mortality, although crucial for understanding treatment efficacy, are influenced by the varied follow-up durations and adjuvant treatment protocols across studies. This variability complicates the direct comparison of outcomes and highlights the challenge of drawing definitive conclusions regarding the optimal surgical approach for LAGC. Additionally, the consideration of English-only studies and potential publication bias toward positive outcomes may limit the review’s comprehensiveness, as important data might be excluded. These factors collectively necessitate a cautious interpretation of the synthesized evidence, emphasizing the need for more standardized, prospective research to validate these findings further.

## 5. Conclusions

Conclusively, multiorgan resection aimed at achieving R0 resection status significantly boosts survival outcomes for patients with locally advanced gastric cancer, although at the expense of heightened surgical risks. This comprehensive analysis firmly establishes the critical role of securing R0 resection in enhancing long-term survival, underscoring the necessity of meticulous surgical planning and the integration of adjuvant therapies tailored to individual patient profiles. The findings advocate for the deployment of specialized surgical skills and a multidisciplinary approach to patient care, emphasizing the importance of personalized treatment strategies. These conclusions not only reinforce the value of MOR in the management of LAGC but also call for ongoing refinement of surgical techniques and postoperative care protocols to optimize outcomes and minimize complications. The study highlights an urgent need for further research to identify predictive markers for surgical success and to refine criteria for patient selection, aiming to ensure that the benefits of aggressive surgical interventions are accessible to those most likely to derive significant survival advantages.

## Figures and Tables

**Table 1:** Study characteristics.

Study and Author	Country	Study Year	Study Design	Study Quality
1 Dhar et al. [21]	Japan	2001	Retrospective cohort	Medium
2 Kobayashi et al. [22]	Japan	2004	Retrospective cohort	Medium
3 Kunisaki et al. [23]	Japan	2005	Retrospective cohort	Medium
4 Carboni et al. [24]	Italy	2005	Prospective cohort	High
5 Kim et al. [25]	South Korea	2006	Retrospective cohort	Low
6 Wang et al. [26]	China	2008	Retrospective cohort	Low
7 Jeong et al. [27]	South Korea	2009	Prospective cohort	High
8 Cheng et al. [28]	Taiwan	2011	Prospective cohort	High
9 Mita et al. [29]	Japan	2012	Retrospective cohort	Medium
10 Pacelli et al. [30]	Italy	2013	Prospective cohort	Medium
11 Xiao et al. [31]	China	2013	Retrospective cohort	Low
12 Li et al. [32]	China	2014	Prospective cohort	Medium
13 Mita et al. [33]	Japan	2017	Retrospective cohort	High
14 Xiao et al. [34]	China	2017	Retrospective cohort	Medium
15 Yang et al. [35]	China	2020	Retrospective cohort	High
16 Dias et al. [36]	Brazil	2020	Retrospective cohort	Medium
17 Aversa et al. [37]	Italy	2021	Retrospective cohort	High
18 Zhang et al. [38]	China	2022	Retrospective cohort	High
19 Bobrzyński et al. [39]	Poland	2023	Retrospective cohort	Medium
20 Vladov et al. [40]	Bulgaria	2023	Retrospective cohort	Medium

**Table 2:** Patient characteristics.

Study Number	Sample Size	Gastric Tumor Location and Features	Follow-Up Time/Mean Survival	Age (years)	Gender Distribution	R0 Resection (%)	Staging, Grading, Histology	Surgery (Excluding Gastrectomy)	Complications	Adjuvant Treatment
1 Dhar et al. [21]	150	Tumor location: NR Gastrectomy type: NR	1 to 3 years	Mean: 62.5 Range: 28–87	Men: 92 (61.3%) Women: 58 (38.7%)	42.1%	T4 gastric carcinoma: 100%	NR	Post-operative complications: 31.3% Post-operative death: 2.0%	Adjuvant: 92.7%
2 Kobayashi et al. [22]	82	Tumor location: NR Gastrectomy type: total 60.9%, subtotal (39.1%)	Median 23.1 months Range 1–93 months	Mean: 64.0 Range: 26–84	Men: 58 (70.7%) Women: 24 (29.3%)	60.9%	T3: 51.2% size 9.0 cm (mean) T4: 48.8% size 10.8 cm (mean)	Pancreatectomy + Splenectomy 43.9% Transverse colectomy 42.7% Liver resection 12.2% Adrenalectomy 8.5%	Post-operative complications: 28.0% Post-operative death: 1.2%	NR
3 Kunisaki et al. [23]	117	Tumor location: lower 37.6%, middle 18.8%, upper 25.6%, entire 18.0% Gastrectomy type: distal 28.2%, total 71.8%	Mean 15.6 months	Mean: 64.7	Men: 77 (65.8%) Women: 40 (34.2%)	32.5%	T4 gastric carcinoma: 100% size 9.1 cm (mean)	Pancreatectomy 27.3% Liver resection 8.5% Transverse colectomy 29.1%	Post-operative complications: 22.2%	No adjuvant therapy
4 Carboni et al. [24]	65	Tumor location: proximal 27.7%, middle 47.7%, distal 21.6%, diffuse 3.0% Gastrectomy type: proximal 1.5%, subtotal 18.5%, total 80.0%	Median 13 months Range 1–163 months	Median: 63 Range: 27–82	Men: 39 (60.0%) Women: 26 (40.0%)	61.5%	T3: 20.0% T4: 80.0%	Splenectomy 47.6% Pancreatectomy 43.1% Colectomy 24.6% Liver resection 18.4%	Post-operative complications: 27.7% Post-operative death: 12.3%	NR
5 Kim et al. [25]	288	Tumor location: upper 12.2%, middle 26.0%, lower 53.8%, diffuse 8.0% Gastrectomy type: total 32.3%, subtotal 62.5%, other 5.2%	1 to 3 years	Mean: 58.0	Men: 198 (68.8%) Women: 90 (31.2%)	32.9%	T4 gastric carcinoma: 100%	Colectomy 58.3% Pancreatectomy 64.9% Liver resection 8.7%	NR	NR
6 Wang et al. [26]	17	Tumor location: NR Gastrectomy type: total 64.7%, subtotal 35.3%	Median 38 months Range 2–72 months	Mean: 56 Range: 38–71	Men: 11 (68.8%) Women: 6 (31.2%)	32.1%	T4 gastric carcinoma: 100% size 4.0 cm (mean)	Pancreaticoduodenectomy 100%	Post-operative complications: 75.0% Post-operative death: 0.0%	Adjuvant: 100% (etoposide + leucovorin +fluorouracil)
7 Jeong et al. [27]	71	Tumor location: distal 36.6%, middle 21.1%, proximal 31.0%, diffuse 11.3% Gastrectomy type: subtotal 33.8%, total 66.2%	Median 17.6 months Range 2.6–44.6 months	Mean: 59.0	Men: 50 (70.4%) Women: 21 (29.6%)	66.2%	T2–3: 36.6% T4: 63.4% size 7.9 cm (mean)	Multiorgan resection: 85.9% Colectomy: 23.9% Pancreatectomy + Splenectomy 46.5% Liver resection: 7.0%	Post-operative complications: 26.8% Post-operative death: 3.3%	Adjuvant: 100%
8 Cheng et al. [28]	91	Tumor location: upper 39.6%, middle 13.2%, lower 38.5%, diffuse 8.8% Gastrectomy type: subtotal 38.5%, total 61.5%	Mean 31.6 months Range 21.9–41.2 months	Mean: 64.2	Men: 62 (68.1%) Women: 29 (31.9%)	81.3%	T4 gastric carcinoma: 100%	Pancreatectomy 59.3% Splenectomy 50.5% Colectomy 26.4% Liver resection 17.6%	Post-operative complications: 28.6% Post-operative death: 4.4%	NR
9 Mita et al. [29]	41	Tumor location: upper 29.3%, 24.4%, 36.6%, diffuse 9.7% Gastrectomy type: proximal 4.9%, subtotal 12.2%, total 82.9%	Median 23.9 months	Mean: 60.0 Range: 43–90	Men: 32 (78.0%) Women: 9 (22.0%)	70.7%	T3: 46.3% T4: 53.7%	Pancreatectomy + Splenectomy 31.7% Pancreaticoduodenectomy 12.2%	Post-operative complications: 17.1%	Adjuvant: 85.4%
10 Pacelli et al. [30]	112	Tumor location: antrum 19.6%, body 40.2%, fundus 18.8%, cardias 8.0%, plastic lynitis 11.6%, gastric stump 1.8% Gastrectomy type: total 67.9%, subtotal 29.5%, degastrogastrectromy 2.7%	Mean 24.9 months	Mean: 63.5	Men: 71 (63.4%) Women: 41 (36.6%)	38.4%	pT4a: 12.5% pT4b: 87.5% Size 7.9 cm (mean)	Colectomy 38.4% Pancreatectomy 41.1% Liver resection 15.2% Splenectomy 3.9%	Post-operative complications: 33.9% Post-operative death: 3.6%	Adjuvant: 100% (epirubicin, cisplatin, fluorouracil)
11 Xiao et al. [31]	63	Tumor location: upper 38.1%, middle 27.0%, lower 23.8%, diffuse 11.1% Gastrectomy type: subtotal 25.4%, total 74.6%	Follow-up mean 13 months Survival mean 19.0 months	56.6	Men: 40 (63.5%) Women: 23 (36.5%)	77.8%	T3: 60.3% T4: 39.7% Poor-undifferentiated: 76.2% Size: 7.2 cm (mean)	Multiorgan resection 50.8%	NR	NR
12 Li et al. [32]	132	Tumor location: proximal 36.2%, middle 20.2%, distal 30.8%, diffuse 12.8% Gastrectomy type: subtotal 28.7%, total 71.3%	5 years	Mean: 58.6 Range: 31–75	Men: 67 (71.3%) Women: 27 (28.7%)	71.2%	T4a: 41.4% T4b: 68.6% Poor-undifferentiated: 78.7% Size: 7.3 cm (mean)	Pancreatectomy 26.6% Colectomy 18.0% Splenectomy 9.6% Liver resection 5.3%	Post-operative complications: 18.1% Post-operative death: 2.1%	Adjuvant: 100% (CapeOX, FOLFOX, SOX)
13 Mita et al. [33]	103	Tumor location: NR Gastrectomy type: NR	Follow-up mean 23 months Survival mean 27 months	Mean 69.7	Men: 81 (78.6%) Women: 22 (21.4%)	82.5%	pT4a: 43.7% pT4b: 56.3%	Pancreatectomy 46.6% Splenectomy 29.1% Colectomy 13.6% Liver resection 11.7%	Post-operative complications: 37.9% Post-operative death: 1.0%	Adjuvant 100% S-1 alone or S-1 and cisplatin
14 Xiao et al. [34]	75	Tumor location: upper 38.1%, middle 27.0%, lower 23.8%, diffuse 11.1% Gastrectomy type: subtotal 25.4%, total 74.6%	Median 32 months	Mean: 56.6 Range: 18–93	Men: 58 (77.3%) Women: 17 (22.7%)	86.7%	T3: 5.3% T4: 94.7% Size: 5.4 cm (mean)	Colectomy 22.7% Liver resection 20.0% Pancreatectomy + Splenectomy 17.3%	Post-operative complications: 9.6% Post-operative death: 0.7%	Adjuvant 69.3%
15 Yang et al. [35]	148	Tumor location: cardia 24.3%, fundus 8.1%, body 27.0%, antrum 29.1%, total 11.5% Gastrectomy type: NR	Median 25.7 months	NR	NR	85.6%	pT3: 9.8% pT4a: 47.1% pT4b: 43.1%	Pancreatectomy 52.9% Splenectomy 56.2% Colectomy 28.1% Liver resection 9.8%	Post-operative complications: 13.1% Post-operative death: 1.3%	Adjuvant 100% (CapeOX, FOLFOX 6–8 cycles)
16 Dias et al. [36]	58	Tumor location: NR Gastrectomy type: subtotal 29.3%, total 70.7%	Median 19.3 months Range 1–106.7 months	Mean: 61.8 Range: 36–81	Men: 41 (70.7%) Women: 17 (29.3%)	87.9%	pT4a: 24.1% pT4b: 58.6%	Pancreatectomy 76% Splenectomy 56% Colectomy 50% Liver resection 24%	Post-operative complications: 53.5% Post-operative death: 8.6%	Neoadjuvant 27.6%
17 Aversa et al. [37]	347	Tumor location: fundus 6.1%, body 13.3%, antrum/pylorus 35.7% Gastrectomy type: total 100%	Median 36 months Range 12–60 months	Median: 65 Range: 45–75	Men: 195/347 Men: 195 (56.2%) Women: 152 (43.8%)	60.8%	pT4b: 100% Size: 7.0 cm (median) Poor-undifferentiated: 82.7%	Multiorgan resection 44.2%	Post-operative complications: 28.6%	Adjuvant radiation 24.5% Adjuvant chemotherapy 43.8% Neoadjuvant chemoradiotherapy 28.5%
18 Zhang et al. [38]	210	Tumor location: proximal 52.4%, distal 41.4%, diffuse 6.2% Gastrectomy type: total 14.3%, subtotal 85.7%	3 to 5 years	Mean: 61 Range: 24–82	Men: 153 (72.9%) Women: 57 (27.1%)	94.3%	pT4b: 100% Poor-undifferentiated: 76.7%	Pancreatectomy 20.5% Colectomy 16.7% Liver resection 9.0%	Post-operative complications: 8.1%	Adjuvant chemotherapy 38.6%
19 Bobrzyński et al. [39]	218	Tumor location: NR Gastrectomy type: total 85%	Follow-up median 101 months Survival median 10.6 months	NR	Men: 153 (72.9%) Women: 57 (27.1%)	46%	cT4b: 100%	Colectomy 18.8% Splenectomy 57.8% Pancreatectomy 23.4%	Post-operative complications: 75%	Neodjuvant 11% (ECF, DCF, and FLOT regimens)
20 Vladov et al. [40]	101	Tumor location: cardia 17.8%, fundus 5.0%, corpus 37.6%, antrum 22.8%, linitis plastica 16.8% Gastrectomy type: NR	Follow-up median 28.1 months	Median: 61 Range: 28–88	Men: 73 (72.3%) Women: 28 (27.7%)	84.2%	T3/T4a: 27.7% T4b 72.3% Poor-undifferentiated: 60.4%	Splenectomy 67.3% Pancreatectomy 32.7% Liver resection 20.8% En bloc resection 73.3%	Post-operative complications: 14.8%	No neoadjuvant

NR—not reported; R0—curative resection with no infiltrated margins.

**Table 3:** Survival analysis.

Study Number	R0 Survival	Non-Curative Survival	Significant Risk Factors (Mortality)	Conclusions
1 Dhar et al. [21]	1 year: 46.7% 3 years: 25.1% 5 years: 16.8%	0% at 2 years	No splenectomy (RR = 2.18): 23.6% vs. 35.0% (splenectomy) at 1 year Esophageal invasion (RR = 2.11): 14.7% vs. 35.5% (no invasion) at 1 year	Pancreatic, double organ, and multiple organ involvement had no effect on patient survival. Splenectomy should be performed along with invaded organ resection.
2 Kobayashi et al. [22]	5 years: 36.9%	0% at 1460 days after surgery	Peritoneal dissemination (RR = 2.22): 21.1% vs. 46.8% (no invasion) at 3 years	Aggressive surgery with curative intent (R0) resulted in a significantly higher survival.
3 Kunisaki et al. [23]	5 years: 32.2%	9.5% at 5 years	Tumor size > 10 cm (HR = 4.79): 0% vs. 55.7% (smaller size) at 3 years Lymph node involvement > 6 (HR = 4.04): 7.0% vs. 55.4% (fewer nodes) at 3 years	Aggressive surgery with curative intent (R0) and lymph node dissection resulted in a significantly higher survival.
4 Carboni et al. [24]	5 years: 30.6%	0% at 5 years	Non-resectability (HR = 3.17)	Aggressive surgical treatment of locally advanced gastric carcinoma with acceptable morbidity and mortality rates improves prognosis, especially when curative resection is achieved.
5 Kim et al. [25]	3 years: 19.9%	5.4% at 3 years	Lymph node involvement (RR = 1.62): 10.1% vs. 21.9% (no involvement) at 3 years	Significant survival benefit of resection (both curative and non-curative) with lymph node invasion and curability as critical prognostic factors.
6 Wang et al. [26]	1 year: 77.0% 3 years: 34.0%	1 year: 41.7% 3 years: 5.6%	NR	En bloc pancreaticoduodenectomy with gastrectomy can improve long-term survival for advanced gastric cancer patients with pancreaticoduodenal region involvement.
7 Jeong et al. [27]	1 year: 74.0% 3 years: 47.5%	1 year: 66.7% 3 years: 15.5%	N3 Lymph node involvement (HR = 5.18)	Aggressive surgery with curative intent (R0) resulted in a significantly higher survival.
8 Cheng et al. [28]	1 year: 60.0% 3 years: 33.3%	1 year: 28.0% 3 years: 0.0%	Liver invasion (RR = 4.49): 17.2% vs. 52.4% (no invasion) at 3 years N3 Lymph node involvement (RR = 9.05)	pT3 patients with MOR had significantly better long-term survival compared to pT4 with MOR and cT4 without MOR. A significantly improved survival for pT4 with R0 MOR resection. Lymph node status, liver invasion, and positive margins were independent prognostic factors.
9 Mita et al. [29]	1 year: 78.1% 3 years: 62.1%	1 year: 28.6% 3 years: 0.0%	Tumor size > 10 cm (HR = 2.87) Non-resectability (HR = 4.46)	Aggressive surgery with curative intent (R0) resulted in a significantly higher survival.
10 Pacelli et al. [30]	5 years: 43.7%	5 years (R1): 31.4% 5 years (R2): 0.0%	Positive peritoneal cytology (HR = 1.35): 16.7% vs. 31.4% (negative) at 3 years N3 Lymph node involvement (HR = 1.83): 21.5% vs. 53.3% (no involvement)	
11 Xiao et al. [31]	1 year: 61.6%	NR	Resectability (HR = 0.33): 68.2% vs. 41.9% (non-resectability) at 1 year Tumor size > 7 cm (HR = 3.58): 35.1% vs. 75.0% (smaller size) at 1 year	Aggressive surgery with curative intent (R0) resulted in a significantly higher survival.
12 Li et al. [32]	1 year: 54.6% 3 years: 22.9% 5 years: 13.8%	1 year: 39.5% 3 years: 7.9% 5 years: 3.5%	N3 Lymph node involvement (HR = 2.49)	Curative resection (R0) with MOR significantly improves survival; lymph node metastasis associated with poorer survival.
13 Mita et al. [33]	1 year: 78.3% 3 years: 47.7%	1 year: 46.6% 3 years: 14.3%	N3 Lymph node involvement (HR = 2.26): 12.1% vs. 51.9% (no involvement) at 3 years Splenectomy (HR = 0.58): 46.7% vs. 40.9% (no splenectomy) at 3 years	Curative resection (R0) with MOR significantly improves survival.
14 Xiao et al. [34]	1 year: 84.8% 3 years: 58.5% 5 years: 34.0%	1 year: 69.8% 3 years: 40.6% 5 years: 29.8%	Linitis plastica (RR = 16.0): 11 months vs. 33 months survival Non-resectability (HR = 15.2): 11 months vs. 34 months survival	Curative resection (R0) with MOR significantly improves survival.
15 Yang et al. [35]	1 year: 59.5% 3 years: 26.0% 5 years: 14.5%	1 year: 27.3% 3 years: 4.5% 5 years: 0.0%	Lymph node involvement > 15 (HR = 2.55): 2.2% vs. 17.1% (fewer nodes) at 5 years Resectability (HR = 2.36): 14.5% vs. 0.0% (non-resectability) at 5 years	Curative resection (R0) with MOR significantly improves survival.
16 Dias et al. [36]	5 years: 56.9%	5 years: 28.6%	Tumor size > 5 cm (HR = 5.76) Lymph node involvement (HR = 13.8)	Curative resection (R0) with MOR significantly improves survival.
17 Aversa et al. [37]	NR	NR	N3 Lymph node involvement (HR = 1.97) Resectability (HR = 1.63): 6.6 months longer survival	Curative resection (R0) with MOR significantly improves survival.
18 Zhang et al. [38]	3 years: 48.2% 5 years: 39.1%	NR	Nerve invasion (HR = 2.21)	Patients received combined resection of pancreas and multiple organs tend to have worse survival.
19 Bobrzyński et al. [39]	Median 15.8 months	Median 7.9 months	Non-resectability (HR = 1.47)	Curative resection (R0) with MOR significantly improves survival but increased the risk of postoperative complications and prolonged hospital stay.
20 Vladov et al. [40]	1 year: 58.3% 3 years: 27.7% 5 years: 18.8%	NR	N3 Lymph node involvement (HR = 5.28) Non-resectability (HR = 4.63)	MOR shows poorer survival and higher complication rates compared to SGRs, but better long-term outcomes than palliative interventions. R0 resection is crucial for better survival, while R1 and N3 status are factors for unfavorable outcomes.

NR—not reported; R0—curative resection with no infiltrated margins; RR—risk ratio; HR—hazard ratio; MOR—multiorgan resection; SGR—standard gastric resection.

## Data Availability

Not applicable.
